# Ocean acidification drives species-specific and time-dependent pigment responses in three phytoplankton taxa

**DOI:** 10.1515/biol-2025-1374

**Published:** 2026-09-03

**Authors:** Emel Kocaman, Koray Ozhan, Mustafa Mantikci

**Affiliations:** Institute of Marine Sciences, Middle East Technical University, Mersin, 33731, Turkiye

**Keywords:** climate change, ocean acidification, phytoplankton, pigment, HPLC

## Abstract

Phytoplankton are primary producers in marine ecosystems and play a central role in biogeochemical cycles, yet their physiological responses to altered seawater pH vary among taxa and over time. Here, we examined the effects of sustained pH manipulation on pigment composition, growth, and dark respiration in three ecologically important phytoplankton taxa – a diatom (*Pseudo-nitzschia* spp.), a dinoflagellate (*Heterocapsa pygmaea*), and a haptophyte (*Emiliania huxleyi*) – during a 15-day controlled laboratory experiment. Cultures were maintained at pH 8.1, 7.8, and 7.5, representing present-day and enhanced acidification conditions. Responses to pH were species-specific and strongly time-dependent. Growth and cell-specific respiration rates showed relatively small and often transient differences among pH treatments, suggesting short-term metabolic adjustment under altered pH. In contrast, pigment composition exhibited clearer and more consistent pH-related responses, primarily expressed as shifts in temporal patterns rather than uniform directional changes. In *Pseudo-nitzschia* spp. and *H. pygmaea*, several key pigments displayed pronounced pH-dependent trajectories, whereas *E. huxleyi* showed greater temporal variability and weaker separation among pH treatments. Overall, these results demonstrate that photophysiological traits respond sensitively to sustained pH changes even when population-level growth and respiration remain comparatively stable, highlighting pigment composition as a potentially sensitive indicator of short-term phytoplankton acclimation to ocean acidification.

## 1 Introduction

Phytoplankton play a central role in regulating marine biogeochemical cycles by controlling primary production and mediating the exchange of climate-relevant gases between the ocean and atmosphere [1]. Atmospheric CO_2_ uptake alters the seawater carbonate system through changes in dissolved inorganic carbon speciation, leading to reduced pH in surface waters, a process widely referred to as ocean acidification [2]. Projections indicate that these chemical changes will intensify over the coming decades, with potential consequences for phytoplankton physiology, community structure, and ecosystem functioning [3]. However, experimental studies have demonstrated that phytoplankton responses to altered pH and CO_2_ availability are highly variable, ranging from enhanced growth to neutral or negative effects, even among closely related taxa [4], [5], [6], [7]. This diversity of responses highlights the strong influence of species-specific physiological traits and environmental context, complicating general predictions of phytoplankton sensitivity to ocean acidification.

Phytoplankton responses to elevated CO_2_ and reduced pH have most commonly been assessed using population-level metrics such as growth rate, biomass accumulation, and changes in community composition. Several laboratories and mesocosm studies have reported enhanced phytoplankton growth under increased CO_2_ availability, often attributed to reduced energetic costs of carbon concentrating mechanisms and increased carbon supply to photosynthesis [4], 5], 8], 9]. In contrast, other studies have documented neutral or negative growth responses under similar conditions, reflecting the combined influence of acid–base regulation costs, nutrient and trace metal availability, and species-specific metabolic constraints [6], 7], 10]. Such contrasting outcomes indicate that growth responses alone do not provide a consistent or sufficient measure of phytoplankton sensitivity to ocean acidification, particularly over short experimental time scales where physiological plasticity rather than long-term adaptation dominates observed responses.

Beyond growth and biomass-based metrics, increasing attention has been directed toward photophysiological traits as sensitive indicators of phytoplankton responses to changing carbonate chemistry. Pigment composition directly influences light harvesting, photoprotection, and cellular energy allocation, and responds dynamically to environmental stressors such as light, nutrient availability, and carbon chemistry [11], [12], [13]. Several experimental studies have demonstrated that elevated CO_2_ or reduced pH can alter pigment concentrations and pigment ratios over short time scales, even in cases where growth responses remain weak or absent [14], [15], [16]. Such changes likely reflect adjustments in photosynthetic regulation and cellular metabolism rather than simple changes in population growth, highlighting pigment composition as a potentially sensitive proxy for physiological plasticity under ocean acidification.

Despite growing evidence that photophysiological traits respond to altered carbonate chemistry, most laboratory studies have focused on short-term experiments spanning only a few days, limiting insight into the temporal evolution and persistence of pigment responses under sustained pH conditions [17]. Moreover, pigment dynamics are rarely evaluated alongside growth and respiration within a unified experimental and statistical framework, making it difficult to assess which physiological traits respond most sensitively to ocean acidification over comparable time scales. In this study, we investigated the effects of sustained seawater pH manipulation on pigment composition, growth, and dark respiration in three ecologically important phytoplankton taxa representing distinct functional groups: the diatom *Pseudo-nitzschia* spp. (Bacillariophyceae), the dinoflagellate *Heterocapsa pygmaea* (Dinophyceae), and the haptophyte *Emiliania huxleyi* (Coccolithophyceae), over a 15-day controlled laboratory experiment. By jointly evaluating multiple physiological traits within a unified statistical framework that explicitly accounts for time-dependent responses, this study addresses a gap in current understanding of how different metrics of phytoplankton physiology compare in their sensitivity to sustained pH manipulation, and whether photophysiological indicators can capture acclimatory signals not reflected in growth or metabolic rate measurements.

## 2 Materials and methods

### 2.1 Experimental setup

The experiment was designed to quantify the effects of seawater pH manipulation on pigment composition, growth, and dark respiration of three marine phytoplankton (the diatom *Pseudo-nitzschia* spp., the dinoflagellate *H*. *pygmaea*, and the haptophyte *E*. *huxleyi*) under controlled laboratory conditions. Stock cultures were maintained at ambient pH (8.1) prior to inoculation. Cultures were not pre-acclimated to the experimental pH treatments, as doing so would have precluded the characterization of time-dependent physiological responses from a common starting point, which was a central objective of this study. The observed responses therefore reflect short-to medium-term acclimatory plasticity under sustained pH manipulation.

Phytoplankton cultures were exposed to three nominal pH treatments: 8.1 (ambient control), 7.8 (moderately reduced pH), and 7.5 (strongly reduced pH). These treatments were selected to encompass present-day surface ocean conditions and elevated acidification levels commonly applied in laboratory studies. Each pH treatment consisted of three independent biological replicates (aquaria) per species.

To minimize biological modification of the carbonate system during incubations, experiments were initiated at relatively low cell densities (approximately 2,000 cells mL^−1^ for *Pseudo-nitzschia* spp., 333 cells mL^−1^ for *H. pygmaea*, and 7,000 cells mL^−1^ for *E. huxleyi*). Experiments for each species were conducted in separate experimental runs to avoid cross-treatment interference; consequently, species-specific responses are interpreted independently rather than statistically compared across taxa.

All incubations were performed for 15 days under a 12:12 h light:dark photoperiod at a photon flux density of 180 µmol photons m^−2^ s^−1^ provided by fluorescent lamps, at a constant temperature of 25 ± 0.2 °C. To prevent nutrient limitation, macro- and micronutrients were added according to f/2 medium concentrations at the start of the experiment. The f/2 formulation provides nutrients at substantially elevated concentrations relative to typical phytoplankton requirements, and experiments were initiated at low cell densities to further minimize the risk of nutrient depletion over the 15-day incubation period. Nutrient concentrations were not monitored or replenished during the incubation, as any addition of fresh medium would have risked disrupting the stability of the carbonate system in the CO_2_-controlled aquaria. Based on prior experience with this medium under comparable experimental conditions, nutrient limitation is not considered to have been a confounding factor. Experiments for each species were conducted in sequential runs separated by approximately one week, during which aquaria were cleaned and sterilized before re-use. Temperature, photoperiod, light intensity, and medium composition were held constant across all runs. f/2 medium was prepared from the same autoclaved filtered seawater stock using a consistent formulation for each run. To reduce potential spatial bias within the incubation chamber, aquaria were positioned randomly rather than grouped by treatment or species.

### 2.2 CO_2_ system and pH manipulations

Surface seawater collected offshore from the Mediterranean Sea was filtered through 0.7 μm glass fiber filters and sterilized by autoclaving (121 °C, 30 min) prior to use. Experiments were conducted in 5 L glass aquaria equipped with pH electrodes, CO_2_ injection lines, and air supply lines. Salinity was measured at the beginning and end of each incubation and remained stable within ±0.1 psu across all treatments.

Target pH levels (7.8 and 7.5) were achieved by controlled bubbling of pure CO_2_ gas into seawater initially adjusted to pH 8.1, while control aquaria were maintained at ambient pH. Each aquarium was connected to an automated monitoring and control system (IKS Aquastar) that regulated CO_2_ input via solenoid valves based on continuous pH measurements. Target pH values were stabilized at least three days prior to phytoplankton inoculation to ensure chemical equilibration.

Throughout the experiment, pH was maintained within ±0.03 units of target values. pH electrodes were calibrated at the start of each experimental run using certified Tris buffer standards prepared in synthetic seawater obtained from Dickson’s Certified Reference Materials laboratory. Reported pH values are given on the total scale. Mean pH values, standard deviations, and ranges for each treatment are provided in the Results section.

### 2.3 Cell abundance and growth rates

Phytoplankton cell abundance was determined using an inverted light microscope (Olympus CX43 equipped with a ToupTek camera) and a Sedgwick–Rafter counting chamber. Samples were gently mixed prior to counting to ensure homogeneity. For each aquarium, cell concentrations (cells mL^−1^) were quantified at each sampling time throughout the incubation period.

Specific growth rates (μ, d^−1^) were calculated over discrete time intervals using the standard exponential growth equation:

$$\mu =\left[\ln\left(X_{2}\right)-\ln\left(X_{1}\right)\right]/\Delta t$$

where *X*_1_ and *X*_2_ are cell abundances at times *t*_1_ and *t*_2_, respectively, and Δ*t* is the time interval in days, and ln denotes the natural logarithm. Growth rates were calculated separately for each growth interval to capture potential phase-specific responses rather than assuming constant growth across the entire incubation.

### 2.4 Respiration rate determination

Dark respiration rates were determined from oxygen consumption measurements conducted under controlled laboratory conditions. For each pH treatment, samples were gently transferred into duplicate 60 mL glass biological oxygen demand (BOD) bottles equipped with optical oxygen sensors, ensuring that no air bubbles were introduced during filling.

All respiration measurements were performed in complete darkness at constant temperature to eliminate photosynthetic oxygen production and minimize diel variability. Incubations were conducted for a minimum of 4 h, during which oxygen concentrations were recorded at one-minute intervals using fiber-optic oxygen sensors (FireSting^®^-O_2_ oxygen meter (PyroScience GmbH)) [18]. Sensors were calibrated prior to each measurement series using a two-point calibration: a zero-oxygen point obtained by sodium sulfite solution and an air-saturated water point at the experimental temperature. The detection limit of the PyroScience oxygen sensors is approximately 0.01 mg L^−1^, which is well below the oxygen changes measured during the incubation periods.

Respiration rates were calculated from the linear decline in dissolved oxygen concentration over time using PyroScience Workbench software. Only incubation periods exhibiting linear oxygen consumption were retained for analysis. Volumetric respiration rates were normalized to cell abundance measured at the time of incubation, yielding cell-specific respiration rates (R_p_, µmol O_2_ cell^−1^ h^−1^).

All respiration measurements were conducted at the same time of day across treatments to ensure consistency. The resulting R_p_ were subsequently analyzed using the statistical framework described above.

### 2.5 Pigment analysis by high-performance liquid chromatography

Phytoplankton pigment composition was determined using high-performance liquid chromatography (HPLC). For each sample, known volumes of culture were filtered onto 25 mm glass fiber filters (GF/F) under low vacuum (<0.5 atm). Filters were stored at −18 °C in darkness until analysis, consistent with standard practice for short-term pigment preservation in laboratory phytoplankton studies.

Pigments were extracted in 90 % acetone (3 mL) and disrupted by ultrasonication for 60 s. Extracts were centrifuged at 5,000 rpm for 10 min, and aliquots of the supernatant were filtered through 0.2 μm syringe filters. Prior to injection, samples were mixed with 1 M ammonium acetate ion-pairing solution.

Pigment separation was performed using an Agilent HPLC system equipped with a C8 column (Thermo Hypersil MOS-2, 150 × 4.6 mm, 3 μm particle size), following the reverse-phase C8 HPLC method described by Barlow et al. [19]. The mobile phase consisted of solvent A (methanol:1 M ammonium acetate, 70:30 v/v) and solvent B (100 % methanol), applied with a gradient elution at a flow rate of 1 mL min^−1^. The gradient program was as follows (time in min; %A:%B): 0 (75:25), 1 (50:50), 20 (30:70), 25 (0:100), and 32 (0:100), followed by column re-equilibration.

The HPLC system was calibrated using commercial standards of major chlorophylls and carotenoids, including chlorophyll *a*, chlorophyll *b*, chlorophyll *c*_*2*_ and *c*_*3*_, fucoxanthin, peridinin, 19′-hexanoyloxyfucoxanthin, diadinoxanthin, alloxanthin, zeaxanthin, lutein, divinyl chlorophyll *a*, and β-carotene. Pigment concentrations were calculated using the external standard method [20].

Pigment concentrations were normalized to cell abundance measured at the time of sampling and expressed as cell-specific values (pg cell^−1^), allowing direct comparison of pigment allocation among species, pH treatments, and sampling times.

Only pigments consistently detected beyond day 0 were included in statistical analyses to avoid inference based on absence-only data.

### 2.6 Statistical analysis

Pigment composition, growth rates, and cell-specific respiration rates were analyzed using linear mixed-effects models (LMMs) to account for the repeated-measures structure of the experimental design. All statistical analyses were conducted in R (version 4.3.2) using the *lme4* package.

For pigment analyses, cell-specific pigment concentrations (pg cell^−1^) were defined *a priori* as the primary response variables. Models were fitted separately for each species and pigment using seawater pH treatment (7.5, 7.8, 8.1), sampling day, and their interaction (pH × Day) as fixed effects, with aquarium identity included as a random intercept. Day 0 samples were excluded from inferential analyses to focus on post-acclimation responses under sustained pH conditions rather than initial transfer effects.

The significance of fixed effects was evaluated using Type III Wald χ^2^ tests. Where significant effects were detected, post-hoc comparisons were performed using estimated marginal means. To account for multiple testing across pigment variables within each species, *p*-values were adjusted using the Benjamini–Hochberg false discovery rate procedure.

Growth rates were analyzed using LMMs with pH and growth interval as fixed effects and aquarium identity as a random intercept. Cell-specific respiration rates were analyzed using the same model structure as pigment concentrations. Model assumptions were assessed by visual inspection of residuals and Q–Q plots, and response variables were log-transformed when necessary to improve normality and homoscedasticity.

Given the limited number of repeated observations per aquarium and uneven temporal spacing following exclusion of day 0, models were specified with random intercepts only to ensure stability and avoid overparameterization. Statistical significance was accepted at *p* < 0.05.

To visualize integrated pigment responses and account for covariance among pigments, principal component analysis (PCA) was applied separately for each species using replicate-level pigment concentration data.

## 3 Results

### 3.1 Growth rate

**Figure 1:**
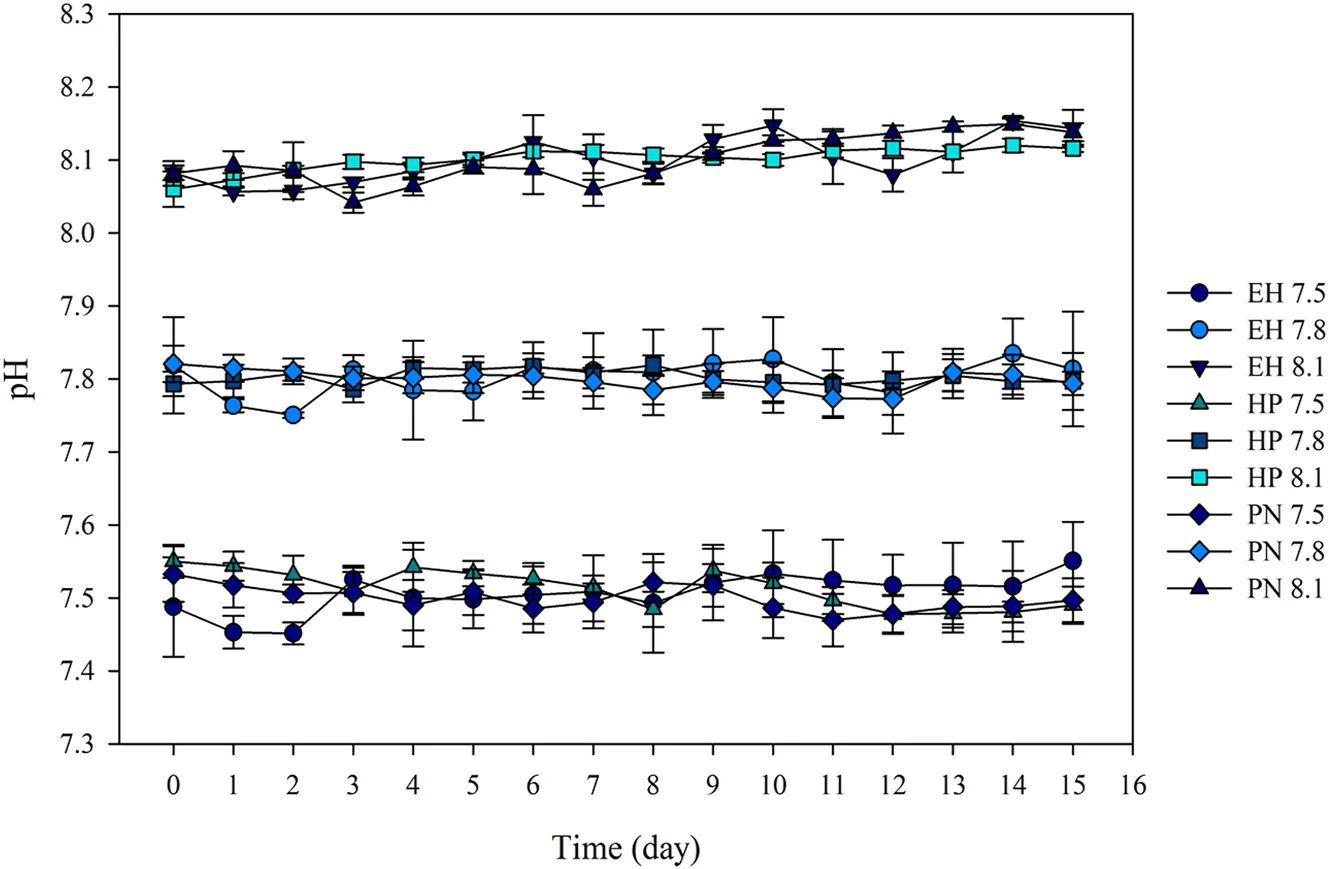
Daily evolution of seawater pH during the 15-day incubations for *(a) E. huxleyi* (EH), *(b) H. pygmaea* (HC), and *(c) Pseudo-nitzschia* spp. (PN) under the three nominal pH treatments (7.5, 7.8, and 8.1). Points show daily mean pH values and error bars indicate ±SD computed from 4–5 replicate measurements per treatment.

Mean seawater pH values were stable and clearly separated among treatments throughout the 15-day incubation for all species (Figure 1). Across experimental runs, average pH (±SD) was maintained at 7.50–7.51 ± 0.02–0.03, 7.80 ± 0.01–0.02, and 8.10 ± 0.02–0.03 for the nominal pH 7.5, 7.8, and 8.1 treatments, respectively.

**Figure 2:**
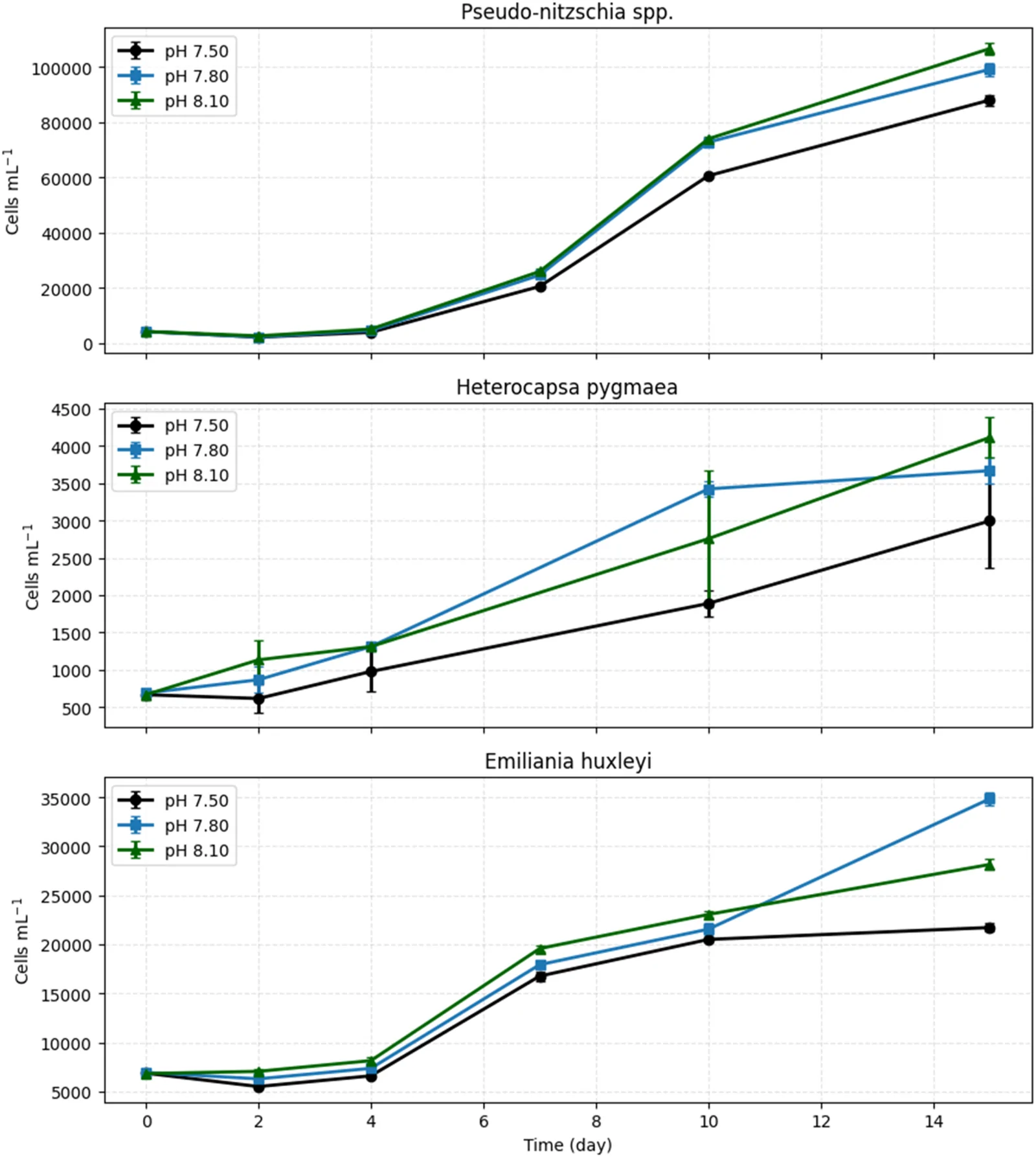
Temporal changes in cell abundance (cells mL^−1^) of *(a) Pseudo-nitzschia* spp., *(b) H. pygmaea*, and *(c) E. huxleyi* during the 15-day incubations under three pH treatments (7.5, 7.8, and 8.1). Points represent daily mean cell abundance and error bars indicate ±SD calculated from replicate cultures.

Temporal changes in cell abundance were broadly similar among pH treatments during the initial phase of the experiment for all species (Figure 2). Following inoculation, cell concentrations increased after day 2, with species-specific differences in growth magnitude and timing. Visual inspection of abundance trajectories did not reveal sustained divergence among pH treatments over the full incubation period.

Specific growth rates calculated over discrete time intervals exhibited limited and phase-specific variation among pH treatments (Table 1). For *H*. *pygmaea* and *E*. *huxleyi*, linear mixed-effects models detected modest differences among pH treatments during the early (day 2–4) and late (day 10–15) growth intervals, whereas no significant pH-related differences were observed during intermediate phases. In contrast, growth rates of *Pseudo-nitzschia* spp. did not show consistent or statistically resolvable divergence among pH treatments across growth intervals.

**Table 1:** Changes in growth rates throughout the experiment for each phytoplankton group; specific growth rate (μ, d^−1^; mean ± SD).

***Pseudo-nitzschia*** **spp.**			

**Interval (day)**	**pH 7.50**	**pH 7.80**	**pH 8.10**

0–2	−0.33 ± 0.04	−0.31 ± 0.02	−0.23 ± 0.02
2–4	0.29 ± 0.04	0.36 ± 0.00	0.33 ± 0.00
4–7	0.55 ± 0.01	0.56 ± 0.01	0.54 ± 0.02
7–10	0.36 ± 0.00	0.36 ± 0.00	0.35 ± 0.01
10–15	0.07 ± 0.00	0.06 ± 0.00	0.07 ± 0.00

***Heterocapsa pygmaea***			

**Interval (day)**	**pH 7.50**	**pH 7.80**	**pH 8.10**

0–2	−0.06 ± 0.21	0.11 ± 0.10	0.26 ± 0.11
2–4	0.24 ± 0.22	0.21 ± 0.12	0.08 ± 0.13
4–10	0.11 ± 0.03	0.16 ± 0.01	0.12 ± 0.05
10–15	0.09 ± 0.03	0.01 ± 0.01	0.09 ± 0.06

***Emiliania huxleyi***			

**Interval (day)**	**pH 7.50**	**pH 7.80**	**pH 8.10**

0–2	−0.11 ± 0.01	−0.05 ± 0.02	0.02 ± 0.02
2–4	0.09 ± 0.00	0.08 ± 0.00	0.07 ± 0.02
4–7	0.31 ± 0.00	0.30 ± 0.02	0.29 ± 0.02
7–10	0.07 ± 0.01	0.06 ± 0.01	0.05 ± 0.00
10–15	0.01 ± 0.00	0.10 ± 0.01	0.04 ± 0.00

Overall, growth responses to altered pH were small in magnitude, temporally restricted, and species-dependent. Across all taxa, no sustained enhancement or suppression of growth was observed under reduced pH conditions over the duration of the experiment.

### 3.2 Respiration rate

**Figure 3:**
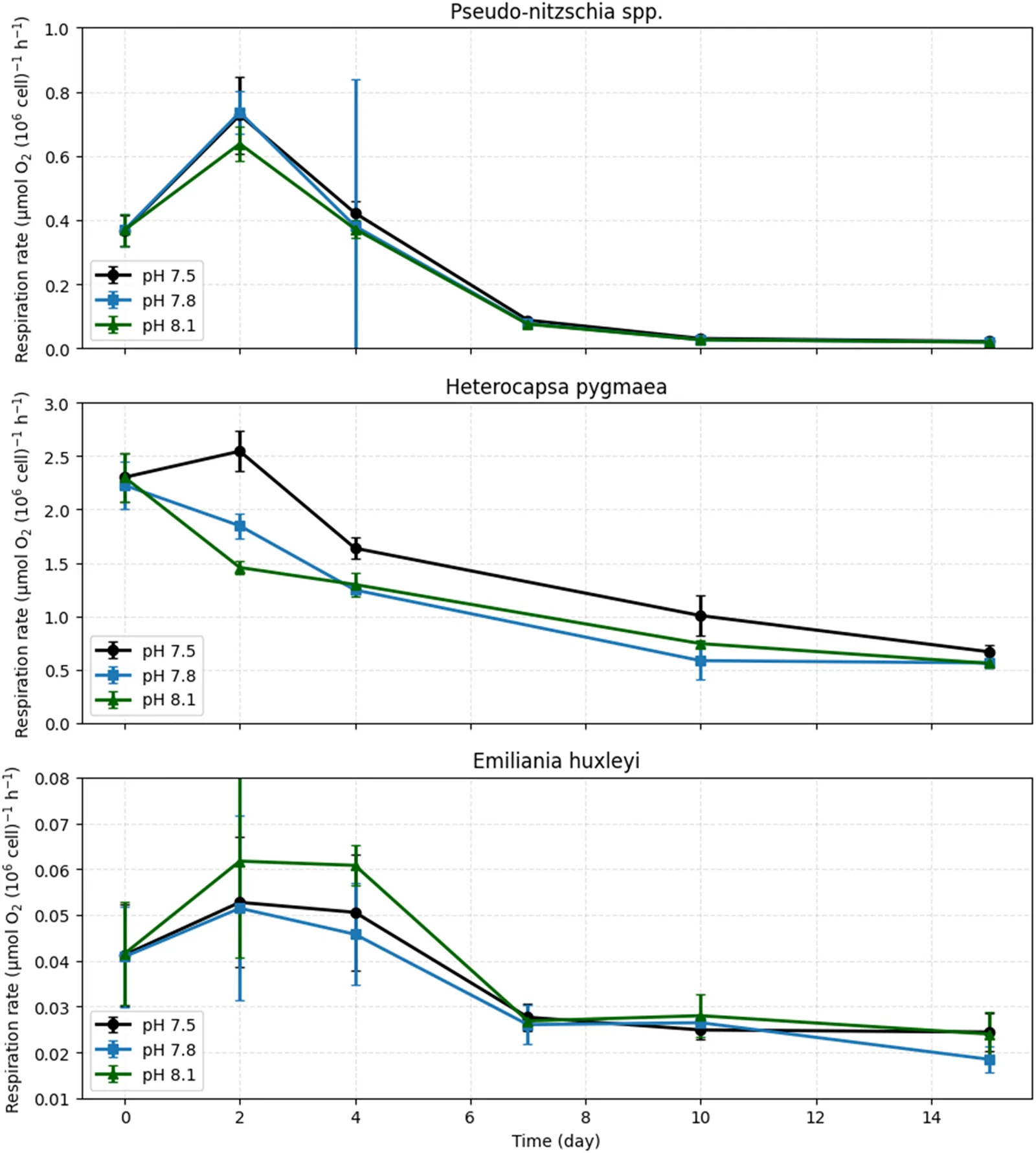
Cell-specific dark respiration rates of *Pseudo-nitzschia* spp., *H. pygmaea*, and *E. huxleyi* under three pH treatments (7.50, 7.80, and 8.10) over the 15-day incubation period. Error bars indicate ±SD calculated from replicates.

R_p_ varied among species and sampling days over the course of the experiment (Figure 3). Across all taxa, temporal variability was the dominant feature of respiration dynamics, whereas differences among pH treatments were comparatively small and inconsistent.

In *Pseudo-nitzschia* spp., respiration rates ranged from low initial values to a transient maximum on day 2, followed by a gradual decline toward the end of the incubation across all pH treatments. No sustained separation among pH treatments was evident over time. Similarly, respiration rates of *H*. *pygmaea* exhibited pronounced temporal variability, with higher overall rates than the other taxa. Under the pH 7.8 and 8.1 treatments, Rp decreased progressively over time, whereas under pH 7.5 an early increase was followed by a subsequent decline. Despite these differences in temporal trajectories, respiration rates did not diverge consistently among pH treatments.

Respiration rates of *E*. *huxleyi* were consistently lower than those of the other species and showed limited variability in magnitude. Maximum R_p_ values were observed during the early phase of the experiment, after which respiration declined across all pH treatments. As for the other taxa, no sustained pH-dependent separation was detected.

Linear mixed-effects model analyses indicated that sampling day accounted for most of the variability in respiration rates, whereas the effect of pH was weak and species-dependent. Across all species, respiration responses to reduced pH were transient and did not persist over the full duration of the experiment.

### 3.3 Pigment composition

Initial pigment composition differed among species at the start of the experiment (Day 0), reflecting known taxon-specific pigment assemblages. *E*. *huxleyi* was dominated by chlorophyll *a*, 19′-hexanoyloxyfucoxanthin, and chlorophyll *c*_*2*_, whereas *H*. *pygmaea* exhibited a more diverse pigment profile characterized by chlorophyll *a*, chlorophyll *c*_*2*_, peridinin, and diadinoxanthin. *Pseudo-nitzschia* spp. was dominated by chlorophyll *a*, fucoxanthin, chlorophyll *c*_*2*_, and chlorophyll *c*_*3*_.

Several pigments detected at day 0 were absent or occurred sporadically at very low concentrations during subsequent sampling days. To avoid inference based on presence–absence patterns rather than repeated-measures variability, only pigments consistently detected beyond day 0 were retained for statistical analyses (Appendix Table 2).

**Figure 4:**
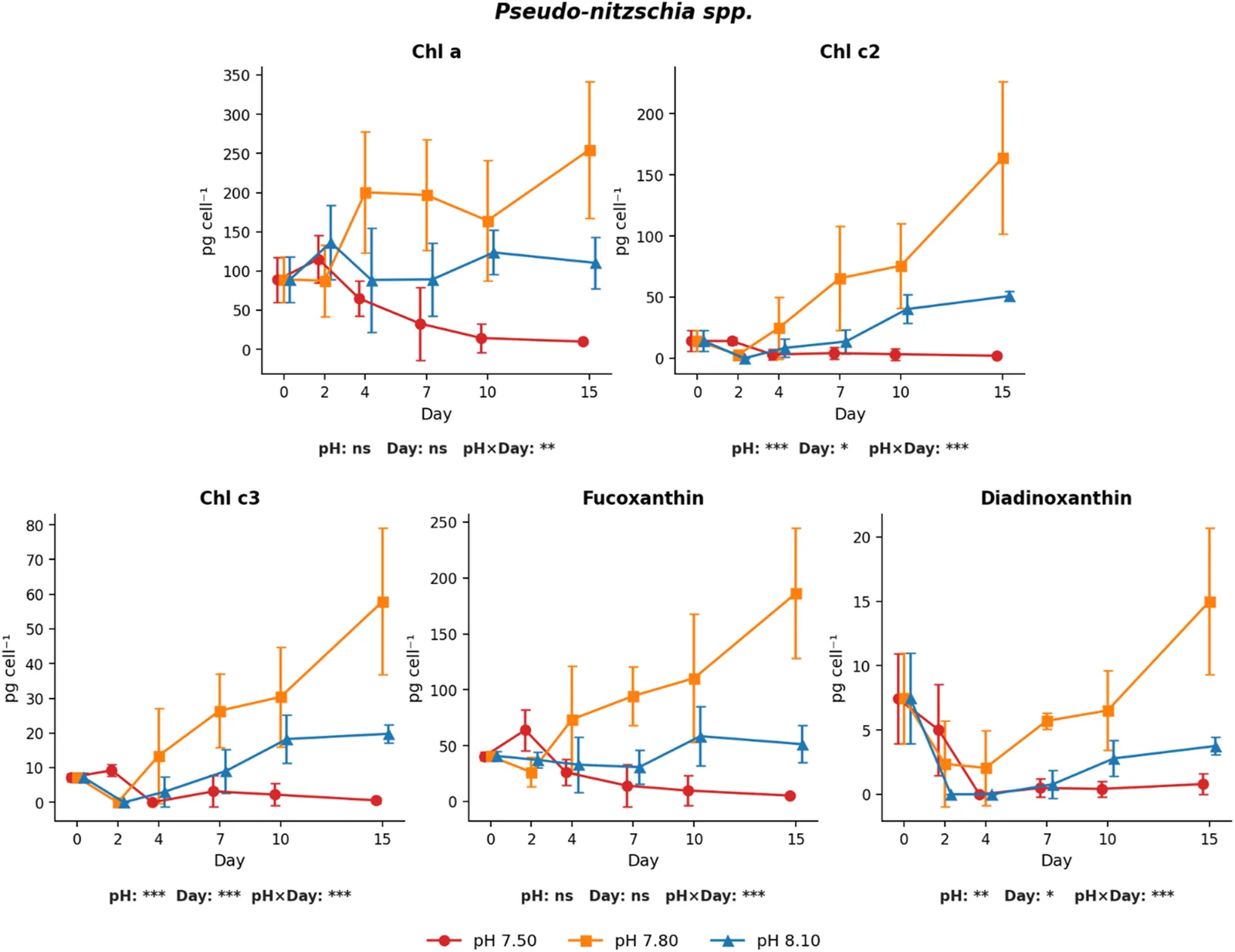
Cell-specific pigment concentrations (pg cell^−1^) of *Pseudo-nitzschia* spp. over time (days 0, 2, 4, 7, 10, and 15) under three pH treatments (7.5, 7.8, and 8.1). Panels show chlorophyll *a* (Chl *a*), chlorophyll *c2* (Chl *c2*), chlorophyll *c3* (Chl *c3*), fucoxanthin (Fuco), and diadinoxanthin (Diadino). Error bars represent ±SD of three biological replicates. Statistical outcomes for pH, day, and pH × day effects are provided in Appendix Table 2.

**Figure 5:**
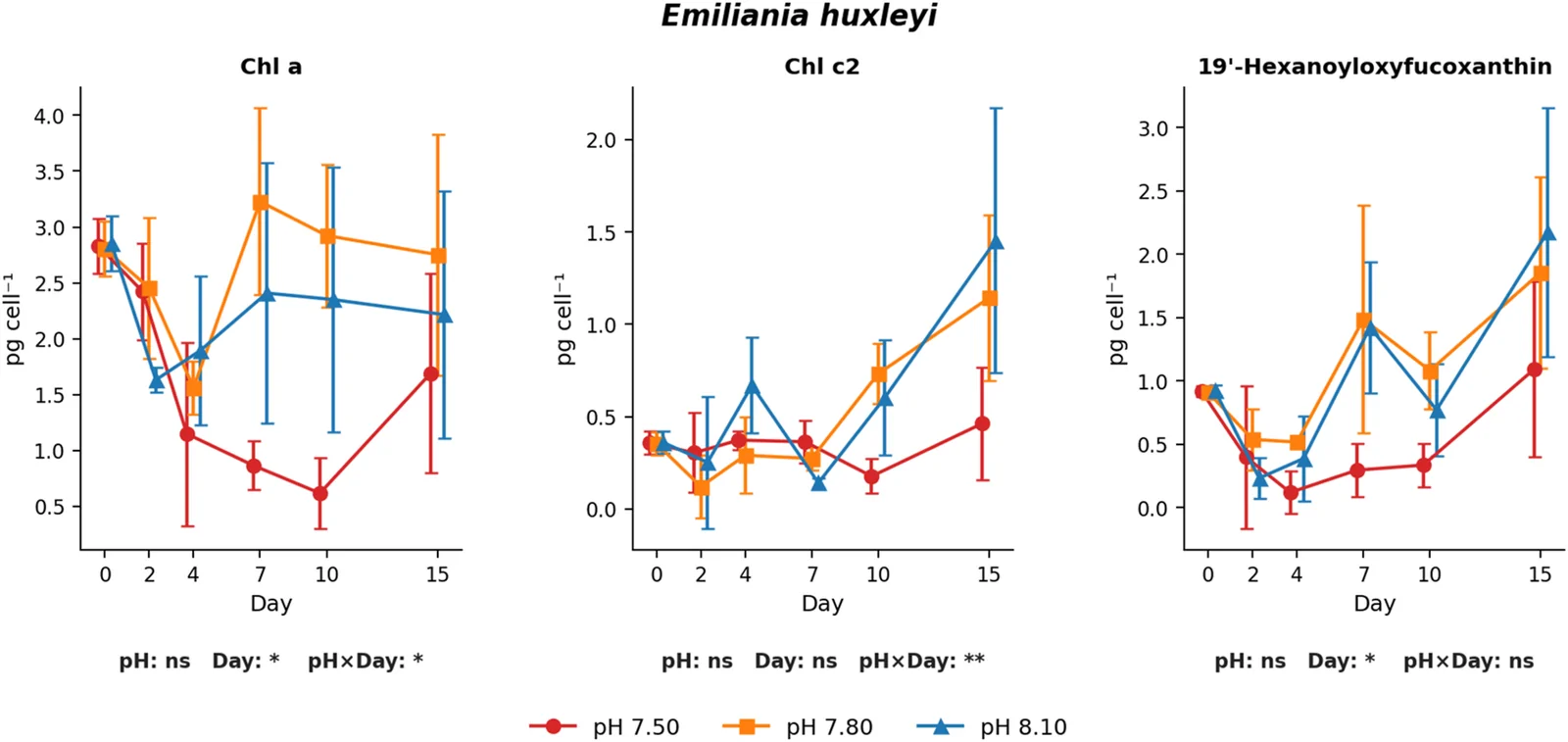
Cell-specific pigment concentrations (pg cell^−1^) of *E. huxleyi* over time (days 0, 2, 4, 7, 10, and 15) under three pH treatments (7.5, 7.8, and 8.1). Panels show chlorophyll *a* (Chl *a*), chlorophyll *c2* (Chl *c2*), and 19′-hexanoyloxyfucoxanthin (19′-Hex). Error bars represent ±SD of three biological replicates. Statistical outcomes for pH, day, and pH × day effects are provided in Appendix Table 2.

**Figure 6:**
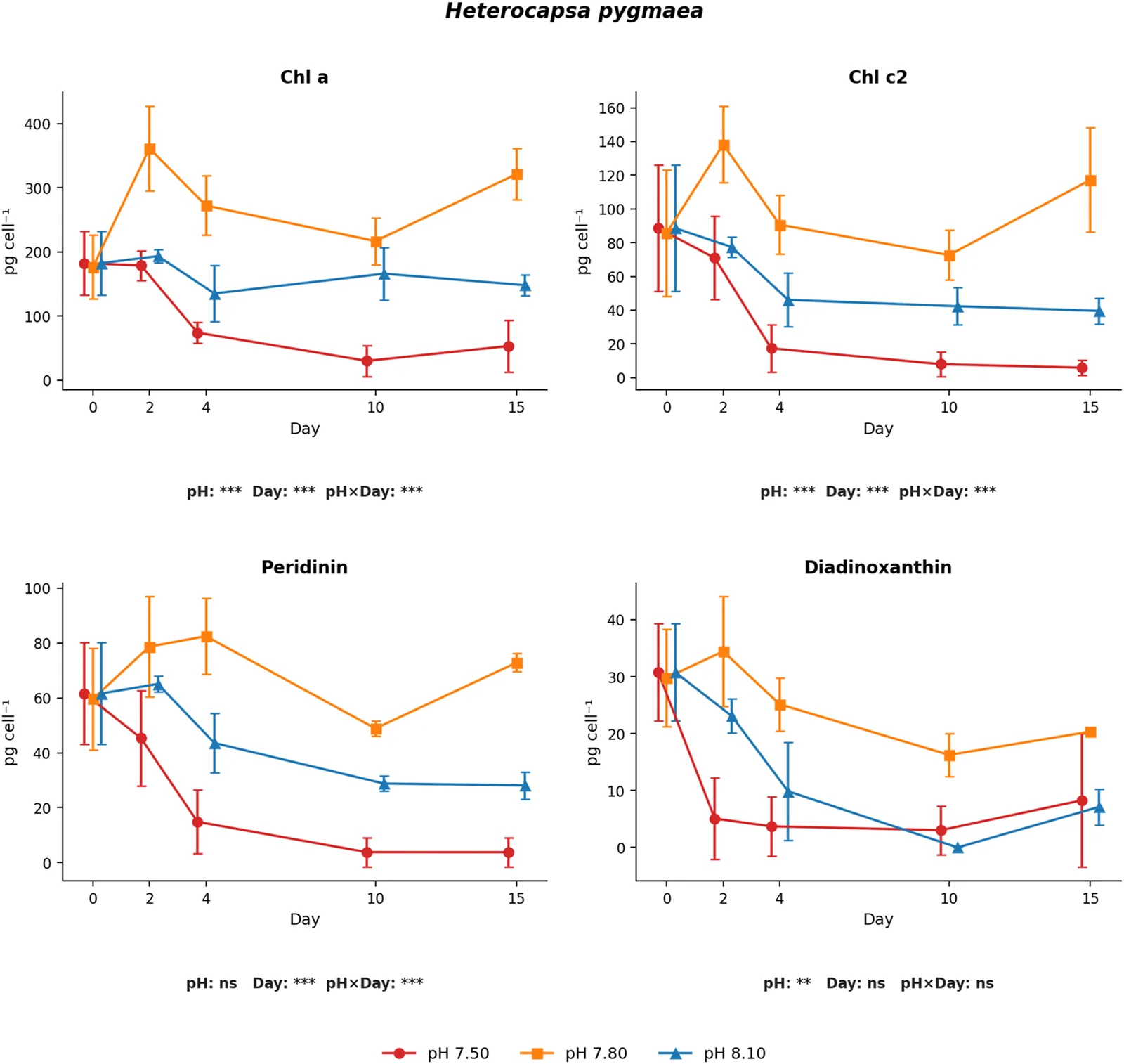
Cell-specific pigment concentrations (pg cell^−1^) of *H. pygmaea* over time (days 0, 2, 4, 10, and 15) under three pH treatments (7.5, 7.8, and 8.1). Panels show chlorophyll *a* (Chl *a*), chlorophyll *c2* (Chl *c2*), peridinin (Peri), and diadinoxanthin (Diadino). Error bars represent ±SD of three biological replicates. Statistical outcomes for pH, day, and pH × day effects are provided in Appendix Table 2.

Across all species, pigment concentrations exhibited pronounced temporal variability over the 15-day incubation (Figures 4–6). Linear mixed-effects models revealed that pigment responses to pH were predominantly expressed as significant pH × Day interactions rather than as uniform main effects of pH (Appendix Table 2), indicating that differences among pH treatments emerged at specific sampling times.

In *Pseudo-nitzschia* spp., multiple pigments, including chlorophyll *a*, chlorophyll *c*_*2*_, chlorophyll *c*_*3*_, fucoxanthin, and diadinoxanthin, exhibited significant pH × Day interactions. Temporal trajectories of these pigments diverged among pH treatments during specific phases of the experiment, whereas consistent directional shifts across the entire incubation period were not observed.

In *E*. *huxleyi*, pigment dynamics were dominated by temporal variability. Chlorophyll *a* and chlorophyll *c*_*2*_ displayed significant pH × day interactions, whereas 19′-hexanoyloxyfucoxanthin showed significant variation with sampling day but limited separation among pH treatments. Overall, differences among pH treatments were weaker and less persistent than those observed for the other taxa.

In *H*. *pygmaea*, pigment responses to altered pH were more pronounced. Chlorophyll *a* and chlorophyll *c*_*2*_ exhibited significant effects of pH, sampling day, and their interaction, indicating substantial divergence in temporal trajectories among treatments. Peridinin showed a strong time-dependent response with significant pH × Day interaction, whereas diadinoxanthin exhibited a significant main effect of pH without a corresponding interaction, suggesting a comparatively consistent sensitivity across sampling days.

Overall, pigment composition responded more consistently to pH manipulation than growth or respiration, with responses expressed primarily as time-dependent modulation rather than sustained directional shifts. The strength and persistence of pigment responses varied among species, with more pronounced pH-dependent temporal structuring observed in *Pseudo-nitzschia* spp. and *H. pygmaea* than in *E. huxleyi*.

**Figure 7:**
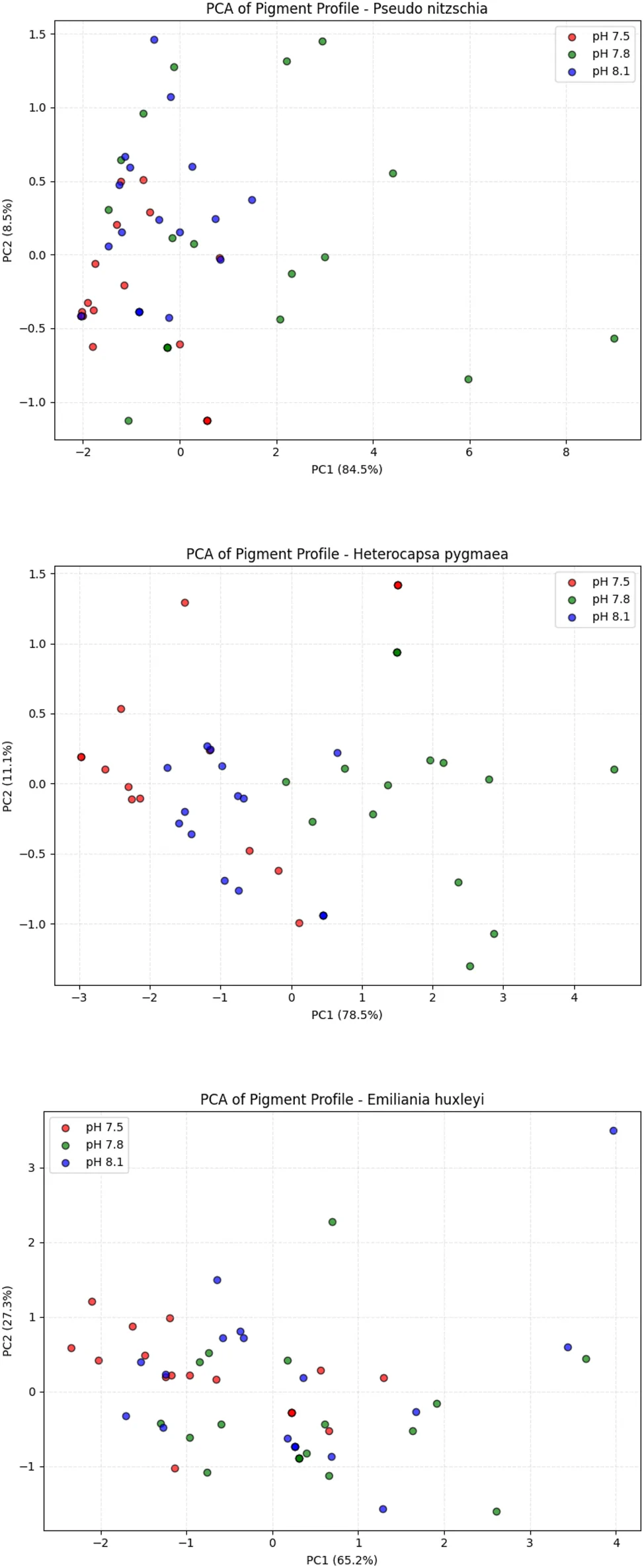
Principal component analysis (PCA) of pigment composition for *Pseudo-nitzschia* spp., *H. pygmaea*, and *E. huxleyi* based on replicate-level pigment concentrations (pg cell^−1^).

To examine integrated patterns in pigment composition and account for covariance among individual pigments, PCA was applied separately for each species using replicate-level pigment concentration data (Figure 7). PCA results were used to visualize coordinated pigment responses rather than for inferential testing. Loading values for PC1 and PC2 for each species are provided in Appendix Table 3.

In *Pseudo-nitzschia* spp., PC1 explained 88.6 % of the total variance. All five pigments loaded positively on PC1 (CHL-A = 0.404, C2 = 0.454, C3 = 0.465, FUC = 0.467, DIAD = 0.442), indicating coordinated variation across the entire pigment complement reflecting overall changes in cellular pigment content. PC2 (7.9 %) was driven primarily by a contrast between CHL-A (loading = 0.832) and the accessory pigments C2, C3, and DIAD (negative loadings), suggesting secondary variation in the balance between the primary light-harvesting pigment and antenna pigments. Samples exhibited partial separation among pH treatments along PC1, with this structure largely reflecting time-dependent variation consistent with the significant pH × Day interactions identified in the univariate mixed-effects analyses.

In *H. pygmaea*, PC1 accounted for 87.0 % of the total variance and showed uniformly positive loadings across all four pigments (CHL-A = 0.521, C2 = 0.514, PER = 0.505, DIAD = 0.458), reflecting global changes in pigment content. PC2 (8.7 %) was dominated by DIAD (loading = 0.879) contrasting with the other pigments, indicating secondary variation in the photoprotective diadinoxanthin pool independent of the photosynthetic pigments. Sample distributions showed temporal structuring with partial differentiation among pH treatments, aligning with the interaction-driven patterns observed in univariate analyses.

In contrast, *E. huxleyi* exhibited weaker structuring by pH treatment in PCA space. PC1 explained 68.1 % of the total variance with positive loadings on all three pigments (CHL-A = 0.552, C2 = 0.518, HEX = 0.653), reflecting coordinated overall pigment variation. PC2 (24.5 %) contrasted C2 (loading = 0.751) against CHL-A (loading = −0.659), indicating secondary variation in the relative allocation between chlorophyll *c*2 and chlorophyll *a*. Samples showed substantial overlap among pH treatments and greater dispersion along temporal gradients, indicating that pigment variability in this species was dominated by time rather than sustained pH-dependent separation.

Overall, PCA results support the univariate analyses by demonstrating that pigment responses to pH manipulation were highly coordinated within species and predominantly time-dependent, with stronger integrated structuring observed in *Pseudo-nitzschia* spp. and *H. pygmaea* than in *E. huxleyi*.

## 4 Discussion

### 4.1 Growth and respiration responses to altered pH

Phytoplankton growth rate is commonly used as a primary indicator of physiological sensitivity to ocean acidification; however, responses reported in the literature span a wide range and often lack consistency across taxa and experimental conditions. In the present study, growth responses to reduced pH were limited in magnitude, transient in time, and strongly species-specific. For *H*. *pygmaea* and *E*.*huxleyi*, modest divergence among pH treatments was restricted to early and late growth phases, whereas *Pseudo-nitzschia* spp. did not exhibit consistent pH-dependent growth differences over the incubation period. These patterns align with previous experimental studies demonstrating that elevated CO_2_ or reduced pH does not universally enhance phytoplankton growth and that growth responses depend strongly on species-specific physiological traits and environmental context [4], [5], [6], [7]. This pattern is further supported by recent observations that short-term acidification exposure leaves growth and community composition largely unaffected even when molecular-level responses are detectable [21], highlighting the disconnect between population-level metrics and underlying physiological adjustments under acute pH stress.

Enhanced growth under elevated CO_2_ has frequently been attributed to reduced energetic costs associated with carbon concentrating mechanisms and increased CO_2_ availability at the site of carbon fixation [22], 23]. However, ocean acidification simultaneously imposes additional physiological demands related to intracellular pH regulation, membrane stability, and ion homeostasis, which may offset potential carbon-related benefits [10], 24]. The absence of sustained growth enhancement across pH treatments in our study suggests that such trade-offs may limit net growth responses under sustained pH manipulation, particularly over short to medium experimental time scales dominated by physiological plasticity rather than evolutionary adaptation.

Respiration dynamics provide complementary insight into these metabolic trade-offs. Cell-specific respiration rates exhibited strong temporal variability across all species, whereas pH-related effects were comparatively weak and inconsistent. Similar patterns have been reported previously, where short-term changes in respiration were attributed to transient acclimation costs rather than persistent metabolic shifts under ocean acidification [25], [26], [27]. The predominance of temporal effects over pH effects observed here suggests that phytoplankton were able to partially adjust respiratory metabolism under sustained pH conditions, resulting in limited long-term divergence among treatments. We acknowledge that bacterial oxygen consumption was not separately quantified in this study. However, as the cultures were non-axenic and bacteria were inseparable from the phytoplankton community, obtaining representative blank respiration measurements was not practically feasible. Any bacterial contribution to oxygen consumption would have represented a uniform background signal across replicates within each treatment rather than a treatment-specific bias, and the absence of significant pH-dependent respiration differences further supports the view that bacterial activity did not systematically confound the results.

Taken together, the growth and respiration results indicate that population-level and metabolic rate metrics alone may underestimate phytoplankton sensitivity to ocean acidification over short to medium time scales. While such metrics are essential for understanding net physiological performance, they may fail to capture more subtle but functionally relevant acclimatory processes that occur without pronounced changes in growth or respiration.

### 4.2 Pigment composition and photophysiological responses

In contrast to growth and respiration, pigment composition exhibited clearer and more persistent responses to pH manipulation across the experimental period. For all three taxa, pigment dynamics were characterized by pronounced temporal variability, with pH effects primarily expressed through significant pH × Day interactions rather than uniform directional shifts. This pattern indicates that pigment responses to altered pH are strongly phase-dependent and reflects dynamic physiological adjustment rather than simple enhancement or suppression of pigment synthesis.

Photophysiological traits, including chlorophyll and accessory pigment composition, are tightly linked to light harvesting, photoprotection, and cellular energy allocation. Changes in pigment pools can therefore capture subtle adjustments in photosynthetic regulation that may not be reflected in population-level growth or respiration metrics [11], [12], [13]. The stronger and more consistent pigment responses observed here suggest that photophysiological traits may serve as informative indicators of short-term acclimation to altered carbonate chemistry, even when net growth responses remain modest, although the predominantly time-dependent nature of these responses warrants caution in generalizing this interpretation [14], [15], [16].

Species-specific differences in pigment responses further highlight the diversity of acclimatory strategies among phytoplankton taxa. In *Pseudo-nitzschia* spp. and *H. pygmaea*, multiple pigments exhibited pronounced pH-dependent temporal trajectories, indicating coordinated adjustments within the photosynthetic apparatus. In contrast, *E. huxleyi* displayed weaker separation among pH treatments and greater dominance of time-driven variability. Such differences are consistent with previous studies showing that pigment responses to ocean acidification can vary substantially among taxa and are modulated by species-specific physiological organization and metabolic strategies [14], 16]. The comparatively weaker responses observed in *E. huxleyi* may partly reflect the unique metabolic constraints associated with calcification, which impose additional energetic demands that modulate photophysiological adjustments under altered carbonate chemistry [28].

The predominance of time-dependent modulation over sustained directional change suggests that pigment responses to altered pH reflect ongoing acclimatory processes rather than fixed physiological states. Under moderately reduced pH conditions, transient increases or stabilization of pigment content were observed during specific experimental phases, whereas stronger acidification was more frequently associated with reduced pigment concentrations at later stages. This temporal structure implies that potential energetic benefits associated with increased CO_2_ availability may be short-lived and increasingly offset over time by physiological costs under lower pH conditions, as suggested by previous experimental work [10], 24].

Importantly, pigment-based responses have broader implications for the interpretation of phytoplankton dynamics in natural systems. Pigment composition underpins widely used approaches for inferring phytoplankton community structure and functional composition, including marker pigment analyses and remote-sensing algorithms [29], [30], [31]. Time-dependent and species-specific pigment adjustments under ocean acidification may therefore complicate the interpretation of pigment-based observations if such physiological plasticity is not explicitly considered.

### 4.3 Experimental scope, limitations, and broader context

The present study was conducted under controlled laboratory conditions with constant pH maintained over the experimental period. This design was intentionally chosen to isolate phytoplankton responses to sustained pH manipulation and to examine time-dependent acclimatory processes without the confounding influence of short-term chemical variability. However, natural marine environments are characterized by pronounced diel and seasonal fluctuations in pH driven by biological activity, physical mixing, and air–sea exchange. Consequently, the responses observed here should be interpreted as reflecting acclimation to sustained pH conditions rather than responses to dynamic pH regimes typically encountered *in situ*.

Carbonate system characterization in this study was limited to pH measurements. Parameters including total alkalinity, dissolved inorganic carbon, and pCO_2_ were not independently measured, which limits direct comparability with studies reporting complete carbonate chemistry. However, pH manipulation was achieved through CO_2_ gas injection, the same process underlying natural ocean acidification, and the primary aim was to examine physiological responses across a defined pH gradient rather than to fully replicate natural carbonate system dynamics. This limitation has been noted in other laboratory studies focusing on photophysiological responses to CO_2_ and pH manipulation (e.g., Sobrino et al. [14]), and its implications for interpretation are acknowledged accordingly.

A further limitation of the present study is the use of three biological replicates per treatment, which is consistent with common practice in controlled laboratory phytoplankton experiments but nonetheless constrains statistical power, particularly for the detection of subtle or moderate physiological effects. It is possible that some treatment-related differences, especially those of small magnitude or restricted to specific sampling intervals, remained below the threshold of detection given the current replication level. Results should therefore be interpreted with this limitation in mind, and future studies employing higher replication would help to confirm and extend the patterns reported here.

The experimental duration of 15 days captures short-to medium-term physiological plasticity but does not address long-term acclimation or evolutionary adaptation. Recent work on *Pseudo-nitzschia multiseries* has demonstrated that short-term experiments can effectively predict long-term physiological responses to combined warming and acidification, suggesting that short-term acclimatory signals identified in laboratory studies may be informative of longer-term trajectories [32]. Previous studies have demonstrated that phytoplankton responses to ocean acidification can change over longer time scales as selection, acclimation, and community interactions become increasingly important [6], 33]. Accordingly, the results presented here provide insight into early and intermediate acclimatory responses but should not be extrapolated directly to long-term population or community-level outcomes.

In addition, the experiment focused on pH manipulation under nutrient-replete and thermally constant conditions, thereby excluding interactions with other environmental drivers such as nutrient limitation, light variability, and temperature. Multi-stressor experiments have shown that such factors can substantially modulate phytoplankton responses to ocean acidification and may amplify or dampen physiological signals depending on environmental context [17]. Future studies integrating dynamic pH regimes and additional stressors will therefore be essential to assess how the pigment-based responses observed here manifest under more realistic environmental conditions.

Despite these limitations, the controlled experimental framework employed in this study offers clear mechanistic advantages. By maintaining stable pH conditions and minimizing confounding variability, the experiment resolves time-dependent physiological adjustments that may be difficult to detect in more complex settings. The results therefore provide a useful baseline for interpreting pigment dynamics under ocean acidification and for guiding future experimental and observational studies aimed at linking photophysiological plasticity to ecosystem-scale processes.

## 5 Conclusions

This study examined species-specific physiological responses of three phytoplankton taxa – *Pseudo-nitzschia* spp., *H. pygmaea*, and *E. huxleyi* – to seawater pH manipulation over a 15-day laboratory experiment. By jointly evaluating growth, respiration, and pigment composition, the results provide a comparative view of which physiological traits respond most sensitively to altered pH conditions over short to medium time scales.

The results demonstrate that photophysiological traits, particularly pigment composition, respond more sensitively to sustained pH manipulation than population-level growth or respiration metrics. While growth and respiration showed limited and transient pH-dependent differences across all taxa, pigment responses were characterized by pronounced temporal structuring, with pH effects expressed primarily through significant pH × Day interactions rather than uniform directional shifts. These findings demonstrate that photophysiological traits can reveal acclimatory responses that are not consistently captured by population-level growth or respiration metrics.

Species-specific differences further underscore the diversity of phytoplankton responses to ocean acidification. Pronounced pH-dependent pigment dynamics in *Pseudo-nitzschia* spp. and *H. pygmaea* contrasted with the more temporally dominated and weaker treatment separation observed in *E. huxleyi*, highlighting the role of taxon-specific physiological organization in shaping sensitivity to altered pH.

Overall, these findings suggest that integrating photophysiological metrics alongside traditional growth- and metabolism-based measurements provides a more complete picture of phytoplankton sensitivity to ocean acidification. The time-dependent and species-specific nature of pigment responses identified here highlights the importance of experimental duration and taxonomic resolution in assessments of phytoplankton acclimation, and points to the value of pigment-based approaches for detecting physiological plasticity in both experimental and field settings.

## Appendix

See Tables 2 and 3.

**Table 2:** Results of linear mixed-effects models (LMMs) evaluating the effects of seawater pH, sampling day (day), and their interaction (pH × day) on pigment concentrations for each species. Models were fitted separately for each species–pigment combination using the formulation pigment ∼pH × day + (1 | aquarium), with aquarium identity included as a random intercept to account for repeated measurements and within-aquarium covariance. Fixed effects were assessed using Type III Wald χ^2^ tests. To control for multiple testing within each species, *p*-values were adjusted using the Benjamini–Hochberg false discovery rate (BH-FDR) procedure.

Species	Pigment	Transform	pH	Day	pH × day
*E. huxleyi*	C2	Log1p	1.09 (2); 0.775 ns	3.52 (4); 0.711 ns	28.58 (8); 0.009**
*E. huxleyi*	CHL-A	Raw	2.14 (2); 0.549 ns	13.69 (4); 0.040*	21.45 (8); 0.036*
*E. huxleyi*	HEX	Log1p	1.26 (2); 0.751 ns	12.14 (4); 0.049*	9.47 (8); 0.522 ns
*H. pygmaea*	C2	Raw	62.36 (2); <0.001***	64.45 (3); <0.001***	66.61 (6); <0.001***
*H. pygmaea*	CHL-A	Raw	89.77 (2); <0.001***	45.79 (3); <0.001***	75.56 (6); <0.001***
*H.pygmaea*	DIAD	Log1p	13.77 (2); 0.001**	0.25 (3); 0.969 ns	7.26 (6); 0.319 ns
*H. pygmaea*	PER	Log1p	3.56 (2); 0.195 ns	51.61 (3); <0.001***	32.81 (6); <0.001***
*Pseudo nitzschia* spp.	C2	Log1p	15.59 (2); <0.001***	11.67 (4); 0.030*	66.23 (8); <0.001***
*Pseudo nitzschia* spp.	C3	Log1p	23.36 (2); <0.001***	24.70 (4); <0.001***	98.95 (8); <0.001***
*Pseudo nitzschia* spp.	CHL-A	Raw	1.31 (2); 0.648 ns	8.31 (4); 0.116 ns	25.49 (8); 0.002**
*Pseudo nitzschia* spp.	DIAD	Log1p	10.92 (2); 0.008**	12.86 (4); 0.019*	35.54 (8); <0.001***
*Pseudo nitzschia* spp.	FUC	Raw	2.68 (2); 0.342 ns	7.99 (4); 0.126 ns	47.68 (8); <0.001***

Analyses were restricted *a priori* to pigments consistently detected beyond day 0, thereby avoiding inference based solely on absence-only data rather than repeated-measures variability. Given the prevalence of significant pH × day interactions, main effects of pH should be interpreted in the context of their temporal dependency; accordingly, pH effects are discussed as time-dependent modulation rather than uniform directional responses across the experimental period. Significance levels are reported as ns (not significant), *p* < 0.05 (*), *p* < 0.01 (**), and *p* < 0.001 (***).

**Table 3:** PCA loadings for PC1 and PC2 for each phytoplankton species. Pigment abbreviations: CHL-A, chlorophyll *a*; *c*2, chlorophyll *c*2; *c*3, chlorophyll *c*3; FUC, fucoxanthin; DIAD, diadinoxanthin; PER, peridinin; HEX, 19′-hexanoyloxyfucoxanthin. Variance explained by each component is given in parentheses.

Pigment	PC1	PC2
*Pseudo-nitzschia spp. (PC1 = 88.6 %; PC2 = 7.9 %)*		

CHL-A	0.404	0.832
C2	0.454	−0.345
C3	0.465	−0.185
FUC	0.467	0.145
DIAD	0.442	−0.365

*Heterocapsa pygmaea (PC1 = 87.0 %; PC2 = 8.7 %)*		

CHL-A	0.521	−0.196
C2	0.514	−0.213
PER	0.505	−0.378
DIAD	0.458	0.879

*Emiliania huxleyi (PC1 = 68.1 %; PC2 = 24.5 %)*		

CHL-A	0.552	−0.659
C2	0.518	0.751
HEX	0.653	−0.038
